# The relationship between nutrition and the immune system

**DOI:** 10.3389/fnut.2022.1082500

**Published:** 2022-12-08

**Authors:** Camelia Munteanu, Betty Schwartz

**Affiliations:** ^1^Department of Plant Culture, Faculty of Agriculture, University of Agricultural Sciences and Veterinary Medicine Cluj-Napoca, Cluj-Napoca, Romania; ^2^Robert H. Smith Faculty of Agriculture, Food and Environment, The School of Nutritional Sciences, The Institute of Biochemistry, Food Science and Nutrition, The Hebrew University of Jerusalem, Rehovot, Israel

**Keywords:** nutrition, health, immunity, nutrients, vitamins, minerals, amino acids, cholesterol

## Abstract

Nutrition plays an essential role in the regulation of optimal immunological response, by providing adequate nutrients in sufficient concentrations to immune cells. There are a large number of micronutrients, such as minerals, and vitamins, as well as some macronutrients such as some amino acids, cholesterol and fatty acids demonstrated to exert a very important and specific impact on appropriate immune activity. This review aims to summarize at some extent the large amount of data accrued to date related to the modulation of immune function by certain micro and macronutrients and to emphasize their importance in maintaining human health. Thus, among many, some relevant case in point examples are brought and discussed: (1) The role of vitamin A/all-trans-retinoic-acids (ATRA) in acute promyelocytic leukemia, being this vitamin utilized as a very efficient therapeutic agent via effective modulation of the immune function (2) The involvement of vitamin C in the fight against tumor cells via the increase of the number of active NK cells. (3) The stimulation of apoptosis, the suppression of cancer cell proliferation, and delayed tumor development mediated by calcitriol/vitamin D by means of immunity regulation (4) The use of selenium as a cofactor to reach more effective immune response to COVID vaccination (5). The crucial role of cholesterol to regulate the immune function, which is demonstrated to be very sensitive to the variations of this macronutrient concentration. Other important examples are reviewed as well.

## Introduction

Food, nutrition and health are highly interrelated and consumption of specific nutrients have a profound impact on human health. The amount and type of nutrients consumed are tightly linked to the metabolic stage and the immune health and thus, inappropriate nutrient consumption is associated with development of major human diseases due to an immune system not properly functioning (1).

The inflammatory mechanisms that compose the innate immunity are strongly influenced by nutrition, and this interaction, when perturbed, can profoundly affect disease development. The immune system is able to destroy antigens through both innate and adaptive immune cells and finally through antibodies that are specific for each pathogen (2).

The number of studies related to the impact of nutrition on immune system is continuously increasing. The initial studies published were related to nutritional-modulation of the immune function were mostly based on the effects of micro and macronutrients (3). Lately, a wide variety of phytochemicals and other chemical biocomponents found in nutrients has been added to the list of nutritional-immuno-modulators. These biocomponents affect the immune function but are not crucial for maintaining normal cell metabolism and function (3). Cases in point are several phytochemicals demonstrated to exert impressive positive immune effects (3).

In light of the strong effects, that we will list in the following paragraphs, nutrients have on the immune system it can be concluded that a rich-nutrient diet is rigorously required in order to maintain an adequate health status. This is in addition to the fact that nutrients are the main factors for survival, including cell proliferation, specialization, development of tissue and organs growth, energy supply, and the immune defense function (4).

**FIGURE 1:**
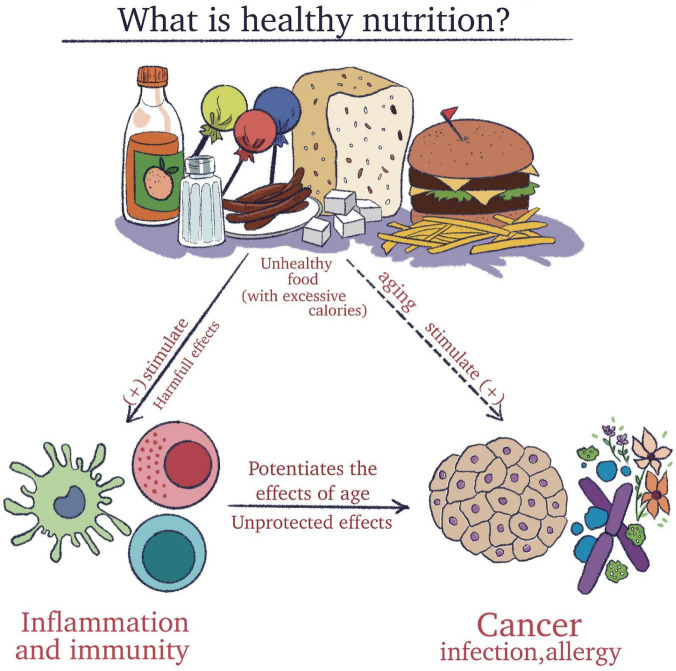
The importance of understanding the meaning of a healthy diet and concomitantly to recognize the harmful effect of pro-inflammatory foods that impact the immune system.

To clarify all these aspects, it is essential to understand the meaning of an adequate diet and concomitantly to recognize the harmful effect of processed foods that impact the immune system (Figure 1).

Moreover, nutritional deficiencies are closely associated with impaired immune response and loss of the host resistance to infection (3). On the one hand, in less developed regions malnutrition continues to be a major health problem (5–7) since it is associated with a higher incidence of morbidity and mortality usually linked with the higher prevalence of bacterial and parasitic infection diseases in these regions (3). In contrast, developed countries confront with inadequate diet consumption, with no real nutritional value, accompanied by excess calories (8). Therefore, malnutrition due to undernutrition or to consumption of poor diets, deficient in macro- and micronutrients, reduce the effectiveness of the immune system, not only by causing a deterioration of the immune protection but also reducing its efficacy in appropriate elimination of the pathogens, thus making people unprotected to a vast variety of diseases.

In addition to food consumption, an important question that arises is regarding the bioavailability of nutrients. Could we treat certain deficiencies using supplements if we are aware of them? Or should we try to build an adequate and complex menu that ensures the proper and desired bioavailability? Another important question refers to where does the absorption of the nutrients take place? It is important to know that the pathogenic agents such as bacterial products, bacteria and some toxic alimentary particles from food and the intestinal microbiota are often responsible for triggering an immune response (9). This is very important because prevention against pathogens is mainly maintained through the intestinal epithelial barrier. The gastrointestinal tract has an essential role due to its lymphoid tissue and for this reason, represents an essential part of the immune system. It has been demonstrated that the epithelial barrier contains cells that present antigens to dendritic cells (DCs) in the lamina propria. The most important are CD103 + CX3CR1- DCs. These imprint the intestinal lymphocytes to stimulate the development of regulatory T cells, the production of IgA, and dendritic cells responsible for the development of Th17 cells and the production of TNFα (10). Additionally, nutrients can control the expression of pro and anti-inflammatory cytokines via interaction with Toll-like receptors (TLRs), which are proteins known to play a key role in the control of the innate immune system. They are located in cells such as macrophages and dendritic cells and as such they control immune cell activity via appropriate crosstalk and signaling. As a result, immune cell’s enzymatic activity is affected and therefore molecular and chemical changes linked to oxidative stress and inflammation take place finally affecting the immune function. Most of the activities are associated with oxidative reactions, affecting neutralized cytotoxicity (11).

The normal standard diets of human beings (except for vegetarians and vegans), include vegetables, eggs, milk, dairy products, and meat. From a biochemical perspective, the foods can be converted into micronutrients and macronutrients that ensure the organism’s well-functioning (12).

This review aims to summarize at some extent the large amount of data accrued to date related to the modulation of immune function by certain micro and macronutrients and to emphasize their importance in maintaining human health.

In order to achieve this goal, research articles and reviews, found in several international databases, have been researched using phrases and keywords. We used the following keywords: nutrition, health, immunity, nutrients, vitamins, minerals, amino acids, and cholesterol.

The review material covers an extended period of time from 1973 to 2022. To achieve this aim some of the issues addressed were: the correlation of nutrition with the immune system in order to obtain good health, certain nutrients involved in the modulation of immune function through mediating pro-and anti-inflammatory responses, and cholesterols’ role in the immune response.

## The effect of nutrition on optimal immune response

As alluded to earlier, nutrition plays an essential role in the regulation of optimal immunological response, by providing adequate nutrients in sufficient concentrations to the immune cells. In such a manner, the immune system can initiate effective responses against pathogens. In order to avoid chronic inflammation, nutrients stemmed from the diet exert significant effects in initiating this quick response (13). When the dietary nutrients are insufficient or inefficient, the supply of these elements to the immune system cells is significantly spared and immunity is compromised.

**FIGURE 2:**
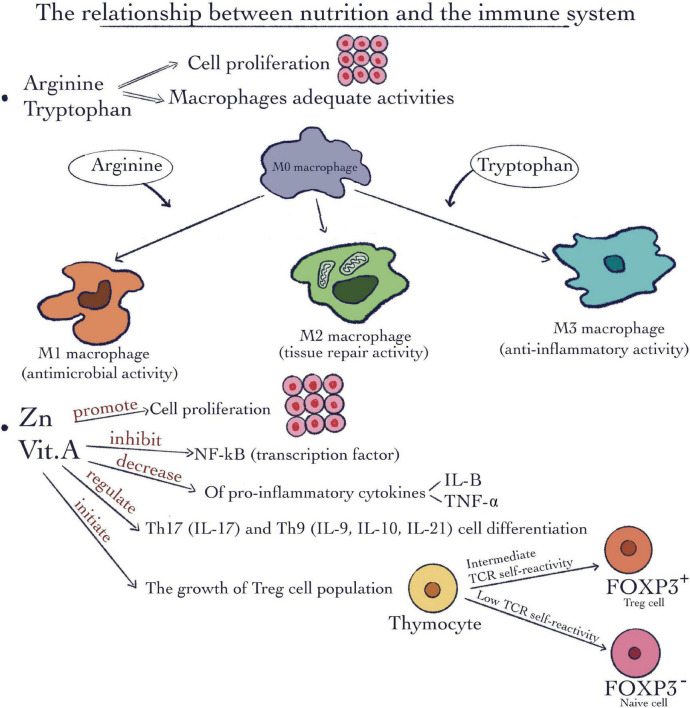
The relationship between nutrition and the immune system. Macronutrients such as arginine and tryptophan are involved in cell proliferation and macrophages’ adequate activities. Micronutrients like Vitamin A and Zinc can promote cell proliferation; inhibit the nuclear factor kappa-light-chain-enhancer of activated B cells (NF-κB) pathway; decrease the pro-inflammatory cytokines IL-1β and tumor necrosis factor-α (TNF-α); regulate the Th17 and Th9 cell differentiation; initiate the growth Treg cell population.

There are certain micronutrients such as vitamins and minerals as well as some macronutrients such as specific amino acids demonstrated to exert a very important and particular impact on immune modulation. Amino acids such as L-arginine and L-tryptophan are responsible and critical for macrophages’ appropriate immune activity. Macrophages are characterized by variations in their plasticity and polarization in response to changes in the intracellular environment. They are capable to transform into different subtypes depending on the intracellular microenvironment and to the different signaling molecules (Figure 2). L-arginine is associated with a well-known immunoregulatory mechanism exploited by M2 macrophages. The mechanism involves arginase 1, which consumes L-arginine and the genes responsible for M1inhibition, concomitantly with M2 promotion (14). Furthermore, arginine and methionine together, are in charge of the synthesis of polyamines. Due to their ability to maintain cell membrane stability and keep DNA homeostasis, they stimulate cell proliferation (Figure 2). In addition, many studies show the involvement of these kinds of amino acids in tumor cell growth metabolic pathways as well as in immune antitumor response (15). Through their degradation, these kinds of amino acids supply chemical precursors for a number of biological reactions (16). Regarding insulin-like growth factor -I and insulin growth hormone these can use as strong secretagogue arginine. Metabolic syndrome and type 2 diabetes as well are major worldwide public health problems which are strongly related to nutrition. There are some amino acids implicated in the synergistic stimulation of insulin release from pancreatic β-cells. One of the most well-known mechanisms is related to the fact that arginine in the presence of glucose can depolarize the plasma membrane at a neutral pH. This gating mechanism is called cationic (17).

Indoleamine 2,3-dioxygenase 1 (IDO1) is a powerful immunosuppressive enzyme involved in the catalysis of the first and rate limiting step of L-tryptophan catabolism. IDO1 depletes L-tryptophan storage and induces the production of immunoregulatory molecules interferon-γ (IFN-γ), tumor-necrosis factor (TNF) and IL-1 (18). High IDO1 expression and catalytic activity occur in dendritic cells (DCs)— in response to IFN-γ (18). Tryptophan metabolism leads to the synthesis of NAD +, which is known as a cofactor capable of redox reactions. In immune tolerance, arginine catabolism may determine the initiation of the nuclear factor kappa-light-chain-enhancer of activated B cells (NF-κB) pathway. Arginine may also provide a substrate for the growth and survival of the cells and concomitantly exerts a key role in differentiation and appropriate gene expression (16).

The decline of protein metabolism that is related to the diminishing concentration of certain amino acids, leads to the endoplasmic reticulum (ER) stress. As a result, the T cells which produce pro-inflammatory cytokines are activated (19). The deficiency of Arg is correlated with reduced T cell ability to trigger tumor immunity (15).

Vitamins and minerals such as vitamin A and Zn in addition to their involvement in cell division and proliferation (Figure 2) are involved in immune-modulation. For example, the rate of antibody synthesis can be modified by these micronutrients (20).

Vitamin A (Figure 2), which is involved in biosynthesis of carotenoids and retinyl esters, molecules well known to affect appropriate immune function (21). It can also exert a role as transcription factor if it is bound to retinoic acid receptors (RARs). As a result, it can be responsible for lipid homeostasis, cell division, growth, and specialization by regulating the expression of certain specific genes (21). Vitamin A deficiency has repercussions on immune functions, such as impaired neutrophil function, suppressing the activity of natural killer (NK) cells, as well as a decline in their number, and damaged capacity of phagocytosing of macrophages. In addition, it may affect the growth and differentiation of B cells (2). In this way, the predisposition for infection disease can increase (22).

Zinc represents another example of the micronutrient group. The transcription factor NF-κB (23) can be inhibited by Zinc (Figure 2). Also, the pro-inflammatory cytokines IL-1β and tumor necrosis factor (TNF-α) production (24) may be repressed, as a result of modulation of the Toll-Like Receptor 4 (TLR4) signaling pathway. Moreover, the pro-inflammatory specific Th17 and Th9 cell differentiation pathway (25, 26) can be moderated by Zn. Treg cell population can be increased after Zinc administration (27, 28) thus Zn is considered an important factor for immune cell development. Specific effects include impaired lymphocyte proliferation, Delayed-Type Hypersensitivity (DTH) response, and natural killer (NK) cell activity (29–31). As we mentioned before, there are strong and dynamic relationships between nutrition and the immune system, which are important for maintaining good health. We will discuss further in more details the role of specific nutrients in the mediation of pro-and anti-inflammatory responses.

## Nutrients involved in mediation of pro-and anti-inflammatory responses

The immune system consists of cells belonging to the two types of immune responses, i.e., the innate and adaptive mechanisms. Once the pathogens enter the body, the first reaction is mediated by the cells belonging to the innate immunity system. This system consists phagocytes, dendritic cells, eosinophils, neutrophils, mast cells, and some additional cells (32). In this case, the immune response acts quickly. The difference between the innate immunity system and the adaptive response is that the former is unspecialized and less efficient (13). In contrast, the adaptive response is capable to recognize each pathogen, and furthermore, remember if it has been encountered before, therefore T cells being the most important in antigen identification. They are also involved in immune response regulation. Furthermore, there are two kinds: cytotoxic T cells/T8 (CD8 inducer), which are implied directly in killing infected cells (33) and tumor cells, and the T4 helper cells (CD4 inducer), which are useful in modulating other cell’s responses. Furthermore, in the function of type cytokines produce by them, there are some subtypes of T helper cells: Th1, Th2, Th17 (13, 34). Th1 cells are responsible for fighting against bacteria and viruses. The main role of these cells is to produce Interferon γ (IFNγ) and IL-2. IFNγ, like IL2, is a cytokine created by both immune adaptive and innate cells like Th1, T8 lymphocytes as well as innate lymphoid cells and NK (35). At the same time, the immune function is activated by Th2 cells. They are capable to produce other interleukins (ILs) (36). Induced cells apoptosis can be caused by activated macrophages and cytotoxic CD8 + T. Interestingly, the other immunity regulatory T cells are involved in the suppression or the blockage of cytokine secretion by the immune response (37). In light of this information, they have a crucial role in peripheral tolerance through the initiation and continuance of this stage (38).

B lymphocytes, which also belong to the adaptive immune system are involved in the synthesis of antibodies. Similar to T cells, they have the ability to specifically respond to each antigen (39). Antigens can actually produce damage to the tissue that they attack. It makes sense that the pathogens in the tissues and around this region promote an inflammatory response. Its main role is to repair the damage tissue in certain ways that can eliminate the antigens and their effects, and decrease their extension (40). After that this process takes place, some physiological changes occur which are responsible for increasing the phagocytes number in the place where this process is happening. As a result, pro-and anti-inflammatory cytokines, prostaglandins, and complements are delivered, especially through the activation of phagocytes. All these changes cause the growth of the inflammatory response (1). Based on the knowledge of which cells are involved in each inflammatory response pathway, it is now feasible to shed light on the effects of specific nutrients on each of these processes.

To this end, we selected to discuss the influence of certain vitamins such as: A, B1, B2, B3, B12, C, and D, minerals like: Zinc, and Selenium as well as certain amino acids such as arginine and tryptophan and some fatty acids.

### Vitamin A

Vitamin A plays an essential role in the regulation of innate and cell-mediated immunity, and antibody responsiveness through the activity of either all-trans retinoic acid, 9-cis retinoic acid or other metabolites and nuclear retinoic acid receptors (41). Vitamin A and associated retinoid metabolites exert an important regulatory function of the immune system. This essential role is evidenced when Vitamin A is deficient and an augmented susceptibility to infections is evident (42). Vitamin A deficiency affects processes related to appropriate cytokines release and antibody production. Additionally, vitamin A deficiency is associated with a reduced production of natural killer cells, monocytes or macrophages, and impaired maturation and proliferation of T- and β-lymphocytes. Vitamin A deficiency impairs innate immunity by impeding normal regeneration of mucosal barriers damaged by infection, and by diminishing the function of neutrophils, macrophages, and natural kill cells. Vitamin A supplementation cuts down morbidity and mortality in various infectious diseases (43). In the case of vitamin A deficiency, the integrity of the mucosal epithelium is altered, resulting in enhanced accessibility to various pathogens to the gastrointestinal tracts and other organs, being children the most affected population (44). In children, severe vitamin A deficiency causes almost the disappearance of goblet cells present in the upper layer of the epithelial line, therefore the production of mucus by these cells is compromised, and bacterial adherence to the epithelial lining is reinforced thus becoming the major factor for the development of the bacterial disease (45). Additionally, vitamin A deficiency is associated with diminished phagocytic activity and macrophage oxidative breakdown that takes place during the process of inflammation along with a reduction in the number of natural killer (NK) cells (42).

It has been demonstrated that vitamin A (Tab.1) stimulates the expansion and differentiation of Th1 and Th2. Thus, vitamin A is capable of promoting the Th2 anti-inflammatory response by repression of IL-12 and IFNγ which are synthesized by Th1 lymphocytes (46). In addition, some studies suggest a positive relationship between vitamin A and mitogen-induced pro-inflammatory cytokine (IFN-γ) and anti-inflammatory cytokine (IL-10) (47). It is important to know that retinol, retinoic acid (RA), and retinal are the three forms of vitamin A. It has been shown that RA is involved in a lot of biological activities (48). According to Rampal et al. (49) under inflammatory conditions, RA might sustain or cause stimulation of intestinal inflammation (50). Moreover, through the release of certain cytokines, such as: IL-1, IL-6, IL-12, and nitric oxide, RA can affect the macrophages’ activity (51). When it comes to hypovitaminosis in children, vitamin A administration reduces mortality caused by diarrheal diseases (52). Vitamin A might be responsible for antitumor effects on human pancreatic cell lines (53). In metastasis of renal carcinoma, it seems that all-trans-RA (ATRA) have a similar effect (54). ATRA represents a nutrient that is required in small quantities and it is synthesized in the human body from the A vitamin (55). In acute promyelocytic leukemia (APL), ATRA is utilized as a very efficient therapeutic agent. Furthermore, together with arsenic trioxide (ATO), they are able to increase life expectancy. Due to this combination, the recovery of this disease is approximately 95% of cases (56). It was observed that, in breast cancer, after the administration of vitamin A, cytotoxic effects have been seen, but the healthy cells weren’t influenced. As a result, vitamin A is capable of reducing some negative chemotherapy effects (57). The next question worth asking would be whether there are more nutrients involved in mediating pro- and anti-inflammatory responses.

### Vitamins B1, B2, B3, and B12

The group of B-vitamins comprise eight water soluble vitamins in charged to carry out essential, inter-related roles for appropriate cellular functioning. These vitamins act as efficient co-enzymes in a vast array of catabolic and anabolic enzymatic reactions and they are essential cofactors for many important cellular metabolic pathways. We therefore cannot refer to life or to cellular life without referring to the B vitamins. In this respect, the crucial enzymes responsible for the regulation of vital functions in cells use specific cofactors such as: Nicotinamide Adenine Dinucleotide/B3, Flavin Mono Nucleotide/Flavin Adenine Dinucleotide/B2, and Thiamine Pyro Phosphate/B1 (58). However, an important aspect to be considered in terms of B vitamins, is that when it comes to the human body, an important source of vitamins B is determined by the activity of the gut microbiota except for some that may be ingested by the diet. The absorption of B vitamins takes place in two different intestinal locations, the large and small intestines. The large intestine represents the main absorption place for most bacterial-produced B vitamins. At the same time, the small intestine represents the place where dietary B vitamins are absorbed. It is tentatively to surmise whether two specific immune responses result from the two different absorption places (59). We surmise that the immune activities at the two specific locations are different since the population of gut immune cells are different (59).

Vitamin B1 or Thiamine (Table 1), exerts an anti-oxidative role due to its protective action on sulfhydryl groups from the surface of neutrophils. As a result, the synthesis of cytokines from macrophages is blocked furthermore. Regarding the stimulation of antimicrobial oxidative reactions myeloperoxidase (MPO), H_2_O_2_, and a halide (HRP/H_2_O_2_/Nal) determined by the activation of polymorphonuclear leukocytes, thiamine together with other compounds can prevent and inhibit this oxidative system of PMNL (60). The NF-κB pathway involved in the control of the oxidative stress, is prevented by Thiamine. This role is highlighted by suppressing the phosphorylation and catabolism of inhibitory kappa B (IκB), which subsequently inhibits the nuclear translocation of the transcription factor-sensitive redox NF-κB (58, 61). From a biochemical and immunological point of view, we can conclude that thiamine derivates are involved in the control of immune metabolism through the regulation of cells’ immune activities. These properties are a result of its function in maintaining an equilibrium between glycolysis and the TCA cycle (62). As we mentioned before, they are cofactors for enzymes participating in these pathway’s. The TCA energy cycle represents the main source of naïve T cells, rest macrophages, and T-regulatory cells. Interestingly, activated T helper cells need energy from aerobic glycolysis because the amount of energy from TCA is not sufficient (63). Due to the significant effects of thiamine on these pathways, B1 deficiencies have so significant side effects. One of the side effects is linked to the stimulation of IL-1, IL-6, and TNF-α (pro-inflammatory cytokines) expression and neuro-inflammation. Finally, neuronal death may occur due to the inhibition of CD 40 and CD 40L regulation (64). It was observed that B1 could be used in the treatment of neurodegenerative diseases through its involvement in the suppression of the pro-oxidative activity of microglial cells (65). Additionally, in regards to B vitamins, we should pay attention to their role in oncogenesis and more over is extremely important to clearly make a distinction between healthy and sick individuals. Some speculations exist regarding the role of B1 in cancer due to its involvement as a cofactor in proliferation and energy pathways that are essential in the development of tumor cells. Further research is needed in order to clearly distinguish Thiamine’s possible oncogenic effects (58, 66).

**TABLE 1:** Vitamins with pro-and anti-inflammatory effects as well as pro-tumor and anti-tumor effects.

Nutrients	Anti-inflammatory effects	Pro-inflammatory effects	Antitumor effects	Tumor effects
Vitamin A	It is capable of promoting the Th2 anti-inflammatory response through repression of IL-12 and IFNγ which are synthesized by Th1 lymphocytes (46). Stimulates production of anti-inflammatory cytokine (IL-10) (47).	It is a positive relationship between vitamin A and mitogen-induced pro-inflammatory cytokine (IFN-γ) (47). Under inflammatory conditions RA may sustain or cause stimulation of intestinal inflammation (50). Through the liberation of certain cytokines such as IL-1, IL-6, IL-12, and nitric oxide is shown that RA may affect macrophages’ activity (51).	It has antitumor effects on human pancreatic cell lines (53). It has antitumor effects in metastasis renal carcinoma by ATRA. It seems that all-trans-RA (ATRA) has an antitumor effect (54). In acute promyelocytic leukemia (APL), ATRA is utilized as a very efficient therapeutic agent (56).	
Vitamin B1(Thiamin)	Anti-inflammatory effects are observed due to the fact that B1 deficiencies side effects is linked to stimulation of IL-1, IL-6, and TNF-α (pro-inflammatory cytokines) expression and neuro-inflammation (64). B1 may be used in the treatment of neurodegenerative diseases through its involvement in the suppression of the pro-oxidative activity of microglial cells (65).			There are some speculations regarding its role in cancer due to its involvement as a cofactor in proliferation and energy pathways that are essential in the development of tumor cells (60, 66).
Vitamin B2 (Riboflavin)	Anti-inflammatory and anti-oxidant modulator, especially in lungs (68, 69). Vitamin B2, act as an anti-inflammatory suppressor. It may block the activation of the NF-κB (74).	B2 bacterial compounds stimulate innate mucosal through invariant T cells which are recognized by their inflammation and defense function in gut mucosal by their products IL-17 and IFN-γ (71).		
Vitamin B3 (Niacin)	Through deacetylation and suppression of NF-κB, NAD may be an anti-inflammatory nutrient (78). It has inhibition effects on inflammatory cytokines (79). It is responsible for diminution of certain alveolar macrophages cytokines such as IL-6, IL-1α, and tumor necrosis factor -α after niacin administration (80). Niacin was considered an inhibitory factor for pro-inflammatory cytokines (80).		It inhibits proliferation of animal tumor cells (79).	
Vitamin B12 (Cobalamin)	It has been found a negative relationship between vitamin B12 and TNF-α (81). Vitamin B12 deficiency is recognized to increase in chronic diseases like insulin resistance (86) and coronary heart disease (87) the inflammatory processes.		No correlation was found between B12 and certain types of cancer like squamous cell carcinoma, prostate, breast, and colorectal (90).	Highintake of B12 was considered hazardous for all types of cancer in a big meta-analysis of cancer patients (92).
Vitamin C	Vitamin C is responsible for preventing activity of pro-inflammatory cytokines and launching the NF-κB reaction (98). In peripheral blood cultures that are stimulated with LPS (lipopolysaccharide) after vitamin C administration was observed an enhancement of IL-10 and a reduction of TNF-α and IFN-γ (99). Vitamin C may be an antioxidant protector for the skin in the fight against ROS as a result of external factors’ synergistic work, particularly pollutants (102).			
	(104) et al., 2015 confirmed the decreased inflammation effects in hypertensive and/or diabetic adults through a moderate decline of inflammatory markers such as hs-CRP and IL-6. It is involved in the regulation of HIF-1α activity, which is capable to make possible neutrophil viability under hypoxic conditions (105).		It is supposed that vitamin C is involved in the fight against tumor culture cells through the number enhancement of the NK cells (107).	
Vitamin D	Vitamin D may be an anti-inflammatory nutrient, through suppression of NF-κB (116). It is responsible for the inhibition of specific pro-inflammatory Th1 cells cytokines like TNF-α, IFN-γ, IL-6, IL-2, and IL-17 (117, 118). It is capable to increase the number of cytokines such as IL-10, IL-4, and IL-5 as a result of an increase in the activity of Th2 cells (119). At a molecular level, through the suppression of pro-inflammatory cytokines and prostaglandins (PG) action as well as stopping the NF-κB signaling pathway calcitriol is considered an anti-inflammatory nutrient (128).		Anti-cancer action of vitamin D is extrapolated in tumor cells by calcitriol which is the active biologically and hormonally compound of vitamin D (127). The stimulation of apoptosis, the suppression of cancer cell proliferation, and delayed tumor development in cancer are certain effects of calcitriol (126, 127). Calcitriol may be used as a preventive and therapeutic agent in cancer (128).	

ATRA, all-trans-RA; HIF-1α, hypoxia-inducible factor 1-alpha; IL, interleukin; IFNγ, Interferon γ; NK, natural killer; NF-κB, pro-inflammatory factor Kappa B; hs-CRP high-sensitivity C-reactive protein; RA, retinoic acid; ROS, reactive oxygen species; Th, helper T cell; TNF-α, tumor necrosis factor.

Riboflavin, or vitamin B2 is crucial for energy metabolism through its function as a cofactor (67). It also plays an important role as an anti-inflammatory and anti-oxidant modulator, especially in lungs (68, 69). Some specific aspects regarding the link between major histocompatibility complex (MHC) and B2 bacterial compounds are worth mentioning. This function on the innate mucosal results in the stimulation of invariant T cells. Riboflavin and its precursors selectively activate mucosa-associated invariant T cells (MAIT) that represent the largest population of innate-like T cells in humans. Their synthesis as well as the link with the major histocompatibility complex through the major histocompatibility complex-protein (MR1) are not fully understood. It was observed that the activation of MAIT cells is dependent on genes that encode enzymes responsible for the formation of intermediate compounds in the synthesis of bacterial riboflavin. (70). These types of cells are known for their function in the inflammation and defense activity in gut mucosal due to their production of IL-17 and IFN-γ (71). The proliferation of neutrophils and monocytes as well as the stimulation of macrophages and neutrophils activities might be boosted by the activity of riboflavin (72, 73). The catabolism of inhibitory kappa B (IκB) is responsible for the activation of the pro-inflammatory factor Kappa B (NF-κB). Following this catabolic pathway, the inflammatory signaling pathway becomes activated. At the end of this signaling pathway, the activation of pro-inflammatory cytokines, such as TNF-α and ILs, takes place. In this signaling process vitamin B2, act as an anti-inflammatory suppressor and it may block the activation of the NF-κB (74). Furthermore, through the overexpression of catalase and nitric oxide synthase vitamin B2 could reduce oxidative stress (75).

Vitamin B3, niacin (Table 1) is known as NADP and NAD precursor. Similarly, to all B vitamins, it is a cofactor for a wide variety of enzymes involved in several metabolic pathways. In contrast to other B vitamin groups, human cells can synthesize NADP and NAD cofactors through independent pathways. From a biochemical point of view, niacin and the resulting cofactors are involved in redox reactions. NAD is responsible for genomic equilibrium and epigenetic regulation may represent its mechanism of action (76). Additionally, there is a positive correlation between high concentrations of NAD and the blockage of ROS synthesis (77). Furthermore, NAD can be considered an anti-inflammatory micronutrient due to its inhibitory and deacetylation actions, which were observed in the NF-κB pathway (78). Also, it has an inhibitory effect on inflammatory cytokines as well as on animal tumor cells (79). NAD is also considered an efficient anti-inflammatory component since it induces the reduction of certain cytokines released from alveolar macrophages (80).

B12, cobalamin (Table 1) affects pro- and anti-inflammatory responses. A negative correlation has been observed between vitamin B12 and TNF-α (81). It has been demonstrated that an increase of TNF-α induce the exhaustion of antioxidants involved in the defense against free radicals (82). As a result, pro-inflammatory cytokines and some other pro-inflammatory compounds are activated (83). Interestingly, in human anemia with cobalamin deficiency, the number of CD8 + T cells decreases compared to the levels in healthy individuals. In contrast, an increase in the number of CD4 + T cells has been observed in patients with cobalamin deficiencies, which differed compared to healthy people. In these cases, the CD4 + /CD8 + ratio is pathological higher. Additionally, in these patients, the activity of NK cells is decreased (84). Interestingly, hyperhomocysteinemia is the result of vitamin B12 deficiency (85), leading to chronic diseases such as insulin resistance (86) and coronary heart disease (87) through the expansion of inflammatory processes. Since vitamin B12 deficiency is associated with abnormal TNF-α activity, it can also lead to insulin resistance (88, 89). Regarding cancer activity, a study by Cheng et al. (90) from a genetic perspective found no correlation between B12 and certain types of cancers such as squamous cell carcinoma, prostate, breast, and colorectal cancer. In the case of lung cancer, B12 administration was not considered a risk factor (91). On the contrary, a higher intake of B12 was considered dangerous for many types of cancer as indicated in a big meta-analysis of cancer patients (92).

### Vitamin C

Vitamin C (Table 1), is considered an essential micronutrient (93) in humans since they cannot synthesize it. Human absorption of vitamin C is higher compared to other species that are capable to synthesized it (94, 95). Vitamin C is involved in the modulation of a wide variety of immune functions and play a role as a regulator of cell-signaling. Vitamin C is also, involved in gene transcription as well as in hydroxylation reactions (96). Through its main function as an antioxidant, it became capable to defend the body against reactive oxygen species that are the result of the activity of toxins and pollution (97).

Vitamin C is responsible for discontinuing the action of the pro-inflammatory cytokines and inhibiting the initiation of the NF-κB reaction (98). In peripheral blood cultures that are stimulated with LPS (lipopolysaccharide), after vitamin C administration, an enhancement of IL-10 and a reduction of TNF-α and IFN-γ has been observed (99). Moreover, as a result of ROS accumulation in microbial infections, vitamin C causes neutrophils displacement into infected sites (100). Additionally, vitamin C might be useful as a cofactor in the synthesis pathways for vasopressin and norepinephrine in severe infections. This has a noticeable effect on the infection response of the cardiovascular system when the pathological state represents a danger (101). It appears that vitamin C may be considered as an antioxidant protector for the skin in the fight against ROS as a result of external factors’ synergistic work, particularly of pollutants (102). In this case, the effect is more pronounced if vitamin C is administrated in combination with vitamin E (103). Ellulu et al. (104) have demonstrated in hypertensive and/or diabetic adults that following C vitamin treatment a decreased inflammation associated with a moderate decline in inflammatory markers such as: the high-sensitivity C-reactive protein (hs-CRP) and IL-6 is observed. Vitamin C is also involved in the regulation of hypoxia-inducible factor 1-alpha (HIF-1α) activity, which makes neutrophil viability under hypoxic conditions possible (105), and in this way, neutrophil apoptosis is delayed (106). Furthermore, it is thought that vitamin C is involved in the fight against tumor cells through the increase in the number of NK cells (107).

### Vitamin D

Vitamin D, (Table 1) exerts many anti-inflammatory roles (108) since receptors to this vitamin are expressed in different organs throughout the human body. The best known and established effects are linked to mineral and bone metabolism (109). Wöbke et al. (108) demonstrated that vitamin D effects are mediated through either nuclear and cytosolic signaling control pathways involving also pro-inflammatory components. Vitamin D binds its receptors (VDR) resulting in a complex of vitamin D-VDR that may contribute to the formation of homodimer with an additional VDR or formation of a heterodimer compound with the nuclear retinoid X receptor (RXR). Also, the nuclear role is demonstrated following the formation of heterodimers with steroid hormone receptors (110). Vitamin D bound to/VDR/RXR can cross the nuclear membrane, and then binds to a response element and start its specific gene regulation action (111) through activation of expression of its responsive genes.

Vitamin D is apparently involved in the adaptive immunity since immune cells such B and T cells express a high number of VDR’s (112). From the immunological regulatory aspect, vitamin D can block the secretion of the pro-inflammatory cytokines IL-6 or TNFα in monocytes (113). Additionally, the same effect has been observed in prostate cells (114). These effects are caused by the inhibition on P-38 MAP kinase (a subclass of mitogen-activated protein kinase) as a response to pro-inflammatory cytokines (113). Moreover, there is an interesting relationship between VDR/RXR and MAP kinase signaling path in terms of activation or inhibition. The results are closely related to the specificity of cells, their response, and effects of triggering factors (115). Furthermore, the complex VDR/RXR can bind to other compounds involved in the transcription process, such as glucocorticoid receptor (GCR) and NF-κB. Thus, vitamin D may be considered an anti-inflammatory micronutrient as a result of these interactions.

The vitamin D bound VDR becomes active and thus exerts inhibitory effects on NF-κB, which is also a heterodimer compound (116). Additionally, some studies suggest anti-inflammatory role of D vitamin is mediated also through the inhibition of specific pro-inflammatory Th1 cell cytokines such as TNF-α, IFN-γ, IL-6, IL-2, and IL-17 (117, 118). Additionally, vitamin D is capable of increasing the concentration of cytokines such as IL-10, IL-4, and IL-5 as a result of an increase in the activity of Th2 cells (119). At the same time, it may induce the amplification of Treg cells as well as a reduction of the number of Th17 cells (120, 121).

From a medical perspective, vitamin D has an important effect on the lung defense system against microbial pathogens. This function represents the result of the antimicrobial peptides activation expression in monocytes, epithelial cells lining the respiratory tract, monocytes, neutrophils, and NK cells (122). Lower levels of vitamin D in the serum are correlated with higher infection risks (123). Particularly, the administration of vitamin D induce a decline in acute respiratory infections (124).

The anti-cancer effect of vitamin D in tumor cells is mediated by calcitriol which is the biologically active molecule of vitamin D (125). The stimulation of apoptosis, the suppression of cancer cell proliferation, and associated delayed tumor development in cancer are the main effects mediated by calcitriol (125, 126). At a molecular level, through the suppression of pro-inflammatory cytokines and prostaglandins (PG) activity as well as by preventing the NF-κB signaling pathway, calcitriol is considered an anti-inflammatory nutrient (127). From this point of view, calcitriol may be used as a preventive and therapeutic agent in cancer (128).

### The minerals-zinc and selenium

When inflammatory cytokines are maintained at a high level, chronic inflammation takes place (129). This process is closely linked to the action of some minerals. In this way, it is important to test what is the role of Zinc in this essential process. The process is mediated (Table 2) by the activity of several signaling pathways that are triggered due to the action of some changes produced by antigens and their metabolites. The main compound involved in inflammatory responses as a result of its role in cell proliferation, cell apoptosis, and the release of certain cytokines like IL-6, IL-8, and IL-1β are mediated by the activity of the NF-κB factor (130). The role of Zinc in this regard is controversial. *In vitro* studies demonstrate that the zinc effects can be either anti-or-pro- inflammatory (131). The NF-κB signaling pathway can be blocked by distinctive intracellular membrane chelator such as TPEN (N, N, N’, N’-tetrakis (2-pyridinylmethyl)-1,2-ethanediamine) (132). From one side the apoptosis effect is evident following the binding of the chelator and heavy metals (133). From the other side, other studies indicate a strong relationship between the initiation of LPS-induced NF-κB and zinc (132). Moreover, after a decline in the release of IL-1β, zinc is able to inhibit pro-inflammatory actions (134).

**TABLE 2:** Selenium and Zinc with pro-and anti-inflammatory effects as well as anti-tumor.

Nutrients	Anti-inflammatory effects	Pro-inflammatory effects	Antitumor effects
Zinc	Zinc may block the NF-κB signaling pathway through chelating with a distinctive intracellular membrane chelator such as TPEN (132). NF-κB signaling pathways may be inhibited by Zinc through a lot of suggested mechanisms (29). Through a decline of IL-1β gene expression, Zinc is responsible for the inflammatory cytokines number reduction. It is capable to inhibit TNF-α (134). It was observed that obese persons with low plasma concentrations of zinc had overexpression of, IL-1β, IL-1α and IL-6 genes (135). The cytokine production is much higher in patients with a critical state of health who were evaluated immediately after intensive therapy due to their decrease in plasma zinc concentration (137). It is able to induce the initiation of the CD8+ T cells proliferation (36, 139)	Some information has shown that the initiation of LPS-induced NF-κB is dependent on zinc (132).	
Selenium	Could be implied in inflammatory mediators production (143, 144). It may acts as a cofactor in immunity that is mediated by the vaccine (148). After selenium treatment was observed a decline in IL-1 and TNF-α gene expression (149). Can enhance the immune response of Th1 cells and the stimulation of T cells (11). Antibody increase titers due to selenium supplementation cause an enhancer of vaccine effects (145, 146).		In patients with cancer, the supplementation of selenium increased antibody titers of IgA and IgG as well as the number of neutrophils (150).

IL, interleukin; IFNγ, Interferon γ; NF-κB, pro-inflammatory factor Kappa B; ROS, reactive oxygen species; Th, helper T cell; TNF-α, tumor necrosis factor; TPEN, N,N,N’,N’-tetrakis (2-pyridinylmethyl)-1,2-ethanediamine.

Furthermore, it has been reported that cytokine synthesis is dependent on Zinc status and this is closely related to chronic inflammation. In this regard, it has been observed that obese people having low zinc plasma concentrations over-express IL-1β, IL-1α and IL-6 genes (135). Zinc exerts beneficial effects on the proliferation and differentiation of T lymphocytes (46). The strong relation between a high number of cytokines and the decline in zinc plasma levels in infections and trauma-associated conditions has been demonstrated in cross-sectional studies. In patients with severe head injuries, upregulated cytokine production genes have been observed (136). In addition, the production of cytokines is elevated in patients that are in a critical state due to their decrease in plasma zinc concentration (137). Moreover, zinc antioxidant effects help the body to defend against reactive nitrogen species (RNS) and reactive oxygen species (ROS) (138). Zinc is able to induce the initiation of the CD8 + T cells proliferation (36, 139). Zinc also can be capable to support the integrity of skin and the mucous membrane (36). To summarize, zinc has an essential effect on the proliferation and development of cells belonging to the immune system, such as T lymphocytes, CD8 + T cells, etc., which are known for their quick turnover. In this regard, it was observed that deficiency of this mineral negatively impacts health by decreasing resistance to infectious diseases, dermatitis, growth diseases, and genetic disorders (140).

Another crucial micronutrient is selenium (Table 2), which is involved in the functioning of the thyroid metabolism and the cardiovascular system as well as in ensuring a functional immune system and preventing cancer. From the cellular point of view, there are still discrepancies regarding the exact dose that may be translated into deficiency or toxicity, even if these stages do not commonly take place in the human body (141, 142). It is well known that when selenium is present within the amino acid selenocysteine is able to control certain metabolic reactions that may lead to lipoxygenase synthesis that finally, can be involved in the production of inflammatory mediators (143, 144). In mice, selenium, due to stimulation of T cell receptor complexes (TCR) activity and conversion of Th1 from T0 cells, may improve the regulation of cellular immunity (145). Selenium can also contribute to the defense against pathogens as a result of its effects on redox signaling activities (146). It was recently demonstrated that in COVID-19 patients, selenium together with zinc exert a protective role and they are associated with a higher chance of survival (147). During vaccination against COVID-19, it has been demonstrated that the response may increase after selenium administration as well as the increase of titers antibodies. It is assumed that selenium may act as a cofactor in immunity response that is mediated by the vaccine (148).

Additionally, in women that are infertile as a result of polycystic ovary syndrome, to whom fertilization *in vitro* has been recommended, a decline in IL-1 and TNF-α gene expression was observed as a result of selenium treatment (149). This effect suggests that selenium has an anti-inflammatory role in the human body. Furthermore, in patients with cancer, the supplementation of selenium increased antibody titers of IgA and IgG as well as the number of neutrophils (150). We can say that selenium is involved in the regulation of the inflammatory mediators’ synthesis and, also, it might increase the activity of phagocytic cells as well. Selenium is capable to enhance the immune response of Th1 cells and the stimulation of T cells. Selenium has a positive relationship with the number of B cells. The innate immune system may be strengthened after selenium administration. A similar effect has been observed on cellular immunity (11). Increased titers of antibodies were measured due to selenium supplements that can cause an enhancement of vaccine effects (145, 146). In the brain, both neurogenesis and hippocampal neural precursor cells are increased after selenium infusion (151).

### Macronutrients: Amino acids arginine and tryptophan

Besides micronutrients, macronutrients, such as proteins and amino acids, also play an important role in the activity of the immune system. Proteins are formed from amino acids that are essential in the construction of other proteins among which antibodies and cytokines that are typical proteins belonging to the immune system (20).

Arginine (Table 3) contribute with the production of nitric oxide in macrophage cells. Nitric oxide (NO) resulting from arginine under the action of nitric oxide synthase (iNOS) determines the cytotoxicity of macrophages in the fight against antigens such as pathogenic bacteria and parasites. Moreover, M1 macrophages use arginine to produce NO (152). Even though that arginine was initially considered a non-essential amino acid (153), after one decade, some papers have proven that arginine is essential for embryonic outliving, ontogenetic fetal development, and for constant hemodynamics and vascular parameters (154). Moreover, the induction of the NF-κB pathway has been linked to the arginine degradation pathway (16). As we presented previously, arginine through cations dependent mechanism can improve the release of insulin from pancreatic β cells. In addition, in β-pancreatic cells, arginine causes an increase in Ca^2+^concentration due to electron transport through a mechanism dependent on the amino acid mCAT2A transporter. When the membrane was depolarized, the Ca^2+^ voltage-dependent channels are opened, followed by the increase in the intracytoplasmic concentration of Calcium and finally the stimulation of insulin secretion. However, clinical evaluations have shown that the beneficial effects of arginine administration are limited, probably due to the fact that it is very quickly transformed into ornithine or citrulline in epithelial cells (17). In addition, the polyamines, compounds which are also derived from arginine degradation, are involved in balanced levels of membrane, mRNA and DNA. Thus polyamines are capable to control the proliferation of cells (155). *In vitro*, polyamines can modify the inflammatory process (156). Furthermore, it has been demonstrated that higher concentrations of intracellular polyamines may change the *in vitro* cytotoxicity regulated by macrophage cells (157). The inflammation regulation and identification of pathogens are closely related to polyamines through their binding manner to receptor-ligand complexes (155).

**TABLE 3:** Macronutrients implied in mediate pro-and anti-inflammatory responses.

Nutrients	Anti-inflammatory effects	Pro-inflammatory effects	Antitumor effects	Tumor effects
Arginine	The induction of the NF-κB pathway was linked with the arginine degradation pathway (16). In Caco-2 cells, arginine was able to induce the inhibition of the IL-1β-mediated NF-κB pathway (160). Some studies that showed the Arg-1 positive effects in certain diseases that are inflammatory through an anti-inflammatory action (162, 163).		There is information that reported the antitumor role of arginine through the improvement of immune response (167).	There are studies were shown that the higher metabolism of arginine in tumors cells together with their particular environment creates conditions that are intermediary and at the same time crucial for the maintenance and development of cancer cells (164, 165). Data were shown that the arginine deprivation is correlated with a delay in the development of some tumor cells (166). (168) et al., 2020 show that certain kinds of cancer cells need arginine to develop. Some authors explained that arginine deprivation may downregulate the migration of cancer cells (169, 170).
Tryptophan (Trp)	IDO-competent cells may trigger an anti-inflammatory action through the Kyn/Trp equilibrium, which has the ability to influence some signaling immune and certain metabolic pathways (182).	Usually, IDO, the tryptophan metabolite has an insignificant effect in healthy and normal conditions. Things are changed by some cytokines, including interferons as a result of the triggered inflammatory process (176). In cases of cancer, infections, auto-immune diseases, or cardiovascular problems the blood ratio Kyn/Trp may represent a marker for inflammation which is linked to IDO activity(181).	The metabolite of tryptophan 5-methoxytryptophan (5-MTP), has the ability to suppress the development of tumors and the displacement of cancer cells in other tissues (187).	The arginine deprivation of cancer cells is capable to reduce metastatic activity (166). Kynurenine metabolism is able to stimulate an oxidative stress resistance pathway, and in this way creating an opportunity to make changes in the tumor microenvironment that help the development of the tumor (186).

Arg-1, type-I arginase; IDO, indoleamine 2,3-dioxygenase; Kyn/Trp, kynurenine/tryptophan; 5-methoxytryptophan, 5-MTP; IL, interleukin; NF-κB, pro-inflammatory factor Kappa B; Th, helper T cell.

In the last decades, it has been discovered that arginine is more beneficial than it has been supposed to be in the past, for example arginine induces the decline of oxidative stress and causes the reduction of the intestine’s inflammation (158).

Arginine is capable of diminishing intestinal damage and reestablishing mucosal immune equilibrium in humans and mammalians’ intestinal diseases (159). In an *in vitro* intestinal system in Caco-2 cells, arginine is able to induce the inhibition of IL-1β-mediated NF-κB pathway (160). However, the mechanism of reducing the inflammatory pathways is still unknown. Perhaps, it is linked to the activity of Arginase-1 (Arg-1), which, in this case, is stimulated by L-arginine as a substrate. The arginase-1 is an enzyme involved in the end of the urea cycle with the aim of forming l-ornithine and urea from l-arginine (161). Some studies suggest that the Arg-1 has positive effects in certain inflammatory diseases through an anti-inflammatory action (162, 163). In contrast, there are studies which have shown that higher metabolism of arginine in tumors cells, together with their particular environment, create conditions that are intermediary, and at the same time, crucial for the maintenance and development of cancer cells. The result of these actions is translated into proper immunosuppression (164, 165). One thing is certain, that the relationship between arginine and cancer cells is controversial. On the one hand there is data suggesting that arginine deprivation is correlated with a delay in the development of some tumor cells (166). On the other hand, arginine can have antitumoral actions which are observed through the enhancement of immune response (167). Furthermore, Al-Koussa et al. (168) have shown that certain kinds of cancer cells need arginine to develop. In this sense, some authors suggest that arginine deprivation may downregulate the migration of cancer cells. In physiological conditions, the movement process is useful for embryonic growth and immune function. But when it comes to cancer cells, things are different. This happens since certain kinds of cancer cells can use this property with the aim to stimulate metastasis (169, 170). Therefore, arginine deprivation in cancer cells is capable of reducing metastatic activity (168). Unfortunately, the exact mechanism remains unknown.

Tryptophan (Trp) is clearly essential for the activity of the immune system (Table 3). Since Trp is necessary for protein synthesis, it becomes to be indispensable for cell division and development (171). Since Trp is not synthesized by the human body, it is required to be obtained from the diet (172). Trp serves as a substrate for the biosynthesis and formation of serotonin (5-HT), kynurenine (Kyn), and indoles (173). The most useful and active Trp metabolism is the Kyn path which is related to the formation of nicotinamide adenine dinucleotide (NAD) and kynurenic acid. Of course, similarly to all pathways, this type takes place due to the involvement of two types of enzymes indoleamine 2,3-dioxygenase (IDO and IDO2) and tryptophan 2,3-dioxygenase (TDO) (174, 175). Additionaly to the Trp metabolism, we brought information on its role in the regulation of inflammation through its initiators, starting with IDO, which exerts an insignificant effect on healthy and normal conditions. Things are changed by some cytokines, including interferons which represent the result of the triggered inflammatory process (176). The highly potent and amply used cytokine interferon-gamma (IFN-γ). It is linked to the promotor-region of IDO and it is capable to express itself in many types of cells. However, the highest expressive grade is found in dendritic cells and macrophages, but there are some other places where it was manifest such as epithelial and connective tissues (177–180). When it comes to cancer, infections, auto-immune diseases, or cardiovascular problems, the serum ratio Kyn/Trp may represent a marker for inflammation which is related to IDO activity (181). As we discussed before, inflammation and chronic immune tolerance are regulated by Trp biochemistry. This being affected by the ability of IDO to change the Kyn/Trp ratio. IDO-competent cells may trigger an anti-inflammatory action through the Kyn/Trp equilibrium, which has the ability to influence some immune signaling and certain metabolic pathways (182). The last step metabolite of the Kyn pathway is represented by the NAD +, which is known for initiating CD8 + and CD4 + lymphocytes programmed death (183). In tumor cells, an important step for metabolic reprogramming is represented by amino acids metabolism. Some authors suggest that, in the case of glioma, there is a strong link between the two because the metabolic amino acid pathway could be used as a predictor for survival as well as certain clinical characteristics (184). As we mentioned before, amino acids and their metabolites are responsible for both controlling malignant cells as well as for changing the microenvironment. In this way, the results are translated into the improvement of immunosuppression and malignancy state (185). Kynurenine metabolism is capable of stimulating an oxidative stress resistance pathway, and, in this way, creating an opportunity to make changes in the tumor microenvironment that helps the development of the tumor (186). However, another metabolite of tryptophan; 5-methoxytryptophan (5-MTP) has the ability to suppress the development of tumors and the displacement of cancer cells in other tissues. Wu et al. (187) consider that these effects are due to suppressing the activity of cyclooxygenase-2 (COX-2). This type of inflammation-associated enzyme is very abundant in tumor cells and also contributes to development process of cancer (187).

## The role of cholesterol in the immune response

Cholesterol has a key function on cellular membranes functionality, especially in the plasma membrane of the cell where it is found at higher concentrations. Its special location at the lipid bilayer allows optimal interaction with other lipids and displays a significant role in membrane fluidity. Cholesterol points its structure mainly into the lipid bilayer leaving only the hydroxyl group facing the external environment. Thus, the steroid rings are in close vicinity to the hydrocarbon chains of adjacent lipids (188). Cholesterol is vital for the many physiological roles that the plasma membrane is involved. The cells keep their lipid bilayer appropriate functionality due to cholesterol molecules, otherwise, microenvironment, endocytosis, signaling pathways, as well as other functions, would be altered. Cholesterol is involved in membrane integrity and it is responsible for receptors arrangement and bilayer fluidity (189, 190). For a better understanding of the information provided later-on, we will describe the cholesterol synthesis and pathway in a schematic frame. Cholesterol biosynthesis of is characterized by a complex pathway, nonetheless the pathways involved have been clearly elucidated (191). Its synthesis involves more than 20 metabolic-specific actions, which include enzymatic reactions belonging to the mevalonate pathway of and additional synthesis pathway of cholesterol. Enzymes involved in cholesterol biosynthesis are mainly detected in the membranes of the endoplasmic reticulum (ER). These enzymes are the target of several molecular reactions which, closely controlled in order to not allow cellular damage (191, 192). However, cholesterol is non-uniformly disseminated in the plasmalemma. In the cell, the plasma membrane represents the main pool/storage. It has been observed that each pool is corresponded to an exclusive function in the plasma membrane physiology (193–195).

It is clear that cholesterol equilibrium involves a transport mechanism by virtue of the concentration gradient from high concentration cholesterol places to regions where cholesterol has been lost or has a low level. The transport of cholesterol is dependent on proteins due to its hydrophobic conformation, thus it cannot be transported through the blood. Thus cholesterol binds to different proteins and forms distinct lipoprotein compounds such as low-density (LDL) and high-density (HDL). As expected, regulatory mechanisms for the formation of each lipoprotein are specific (196). The surplus of cholesterol can be transported through the efflux process or deposited as intracellular lipid droplets (191) because of the incapacity of most human cells to efficiently degrade it. The deposition of lipid droplet plaques in the bloodstream causes the release of inflammatory cytokines which create later an inflammatory process. The consequence of this event is associated with inflammation triggered by the cytokine interleukin-1β (IL-1β) (197). Furthermore, IL-1β is considered an important marker in the inflammatory process (198).

**FIGURE 3:**
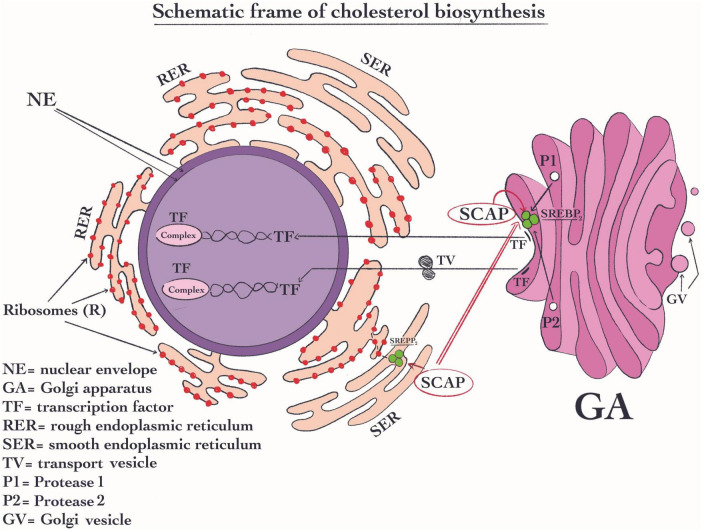
Schematic frame of cholesterol biosynthesis. In the signaling pathway of cholesterol, sterol response element-binding protein SREBP2 has an essential role. SREBP2 is located in RE, where it forms a complex with the protein like cleavage-activating protein and generating (SCAP); Its most important activity is to reduce the cellular cholesterol concentration when this is higher. Then, SCAP is shifted from ER to the Golgi apparatus. Once SCAP reaches the Golgi apparatus, proteases sit 1 and 2 digest this complex and subsequently, the transcription factor (TF) is formed and is activated (202). Then, TF moves into the nucleus where it becomes active and control the transcription of the enzymes of cholesterol biosynthetic pathway.

The cholesterol signaling pathway plays a role in the immune response we therefore will highlight these pathways. Sterol response element-binding protein (SREBP) exerts an essential role in the signaling pathway of cholesterol (Figure 3). Normally, these proteins are located in the membrane of the ER, which is capable of binding with additional two complex proteins such as the cleavage-activating protein and generating (SCAP) (199) and the insulin-inducible genes (INSIGs) (200). The shift of SCAP from ER to the Golgi apparatus plays a key role in its activity. SREBPs proteins are composed of three variants SREBP1a, SREBP1c, and SREBP2, being the latter the most important (199). SREBP2 is a protein complex structure that seems to be capable to regulate the expression of all the enzymes that are involved in cholesterol biosynthesis (201). Its most important activity is its specific response to high concentration of sterols which are able to efficiently induce a decrease in cholesterol synthesis. SREBP2 fulfills its function when the sterols concentration decrease. This change in sterol concentration due to SREBP2 activity will generate afterward the shifting the complex SCAP from ER to the Golgi apparatus (200). In this organelle, the SCAP molecule is changed (199). Once the SCAP reaches the Golgi apparatus, proteases sit 1 and 2 cut this complex (Figure 3). As a result, the transcription factor (TF) is created and stimulated (202). Then the TF enters the nucleus where it is responsible for the regulation of the cholesterol synthetic pathway enzymes (203). Regarding immune-related mechanisms, SREBP2 may be activated in the immune cells’ T cell receptor (TCR) and B cell receptor (BCR) through their signaling pathways. All these pathways may stimulate the flux of cholesterol biosynthesis (204, 205).

**FIGURE 4:**
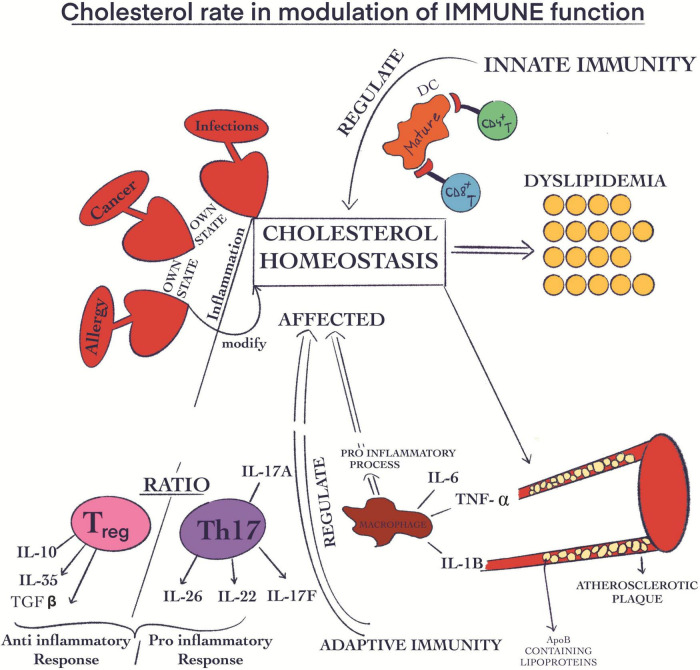
Cholesterol rate in the modulation of immune function. Some diseases, such as cancer, infections, and allergies, are capable of modifying the cholesterol biochemical equilibrium through their own state of inflammation; Atherosclerosis and dyslipidemia are negative effects of the increased cholesterol biosynthesis; The ApoB-containing lipoproteins are accumulated in endothelial place; This phenomenon has pro-inflammatory effects; The inflammation is correlated with the generation of certain cytokines like (TNF) α, IL6, and (IL)1β; The Treg cells have an anti-inflammatory effect; Th17 cells are involved in the pro-inflammatory process; The decline of Treg/Th17 ratio is linked with long-term inflammation.

Additionally, it is important to mention that all cholesterol associated pathways involving synthesis, influx, efflux, and esterification take place through mechanisms closely related to each other allowing well-adjusted whole mechanistic biochemical pathways. All these tightly controlled mechanisms highlight the crucial role of cholesterol in life span, and clarify the potential risks when the concentrations are diverted from the optimal range. In this regards, Luo et al. (191) have demonstrated that there is a correlation between certain metabolic diseases such as familial hypercholesterolemia, Schnyder corneal disease and altered cholesterol metabolism (191). Moreover, in several diseases such as various types of cancer, infections and allergies, cholesterol biochemical equilibrium is severely altered through inflammation-associated consequences. Regarding the relationship between cholesterol and macrophages, counter-regulatory mechanisms oppose macrophage inflammation and at the same time cholesterol cellular accumulation. When the concentration of cellular cholesterol increases, specific sterols are formed. With their help, the transcription factors liver X receptor (LXR)–retinoid X receptor (RXR) are activated. These heterodimers have anti-inflammatory roles, including controlling the expression of ATP-binding cassette transporters (ABC transporters), which are ABC subfamily A member 1 (ABCA1) and ABCG1. They are also involved in stimulating the efflux of cholesterol from macrophages. In this way, they can suppress the activation of TLR signaling given by the increased intracellular cholesterol concentration. When TLRs are activated, LXR genes are inhibited, thus decreasing the cholesterol efflux from macrophages. Activation of cholesterol efflux by ABCA1 and ABCG1 is via apolipoprotein A1 (APOA1), forming HDL and initiating the process of transporting cholesterol back to the liver via blood vessels and lymphatics. Therefore, as a way of amplifying the inflammatory response, the immune system can alter cholesterol homeostasis (206). When the control of cholesterol biosynthesis is disturbed resulting in high cholesterol levels it can be translated into metabolic diseases such as atherosclerosis and dyslipidemia (Figure 4). In some of these cases, both the innate and the adaptive immune functions have the ability to regulate this phenomenon (207). In this way, ApoB-containing lipoproteins are originated immediately after atherosclerosis damages. These are generated, developed and stored in the endothelial compartment (208). Interestingly, these molecules exert pro-inflammatory effects through acetylation, oxidation, and especially induce aggregation with additional molecules (209). The modifications provoked by the accumulation of ApoB-containing lipoproteins (Figure 4) in endothelial location results in the growth of adherence, hold, and mobility in this place of immune cells (210). In summary, the inflammation is supported through the generation of ROS and certain cytokines such as (TNF) α, IL6, and (IL)1β (208, 210). When it comes to the inflammatory stage, the Treg cells exert an anti-inflammatory effect through the inhibition action of CD8 + Th and CD4 + T cells. At the same time, Th17 and Th1 are involved in the pro-inflammatory process (209, 211). Long-term inflammation (Figure 4) and the development of atherosclerosis are strongly linked with the decline of the Treg/Th17 ratio (212–214).

## The polyunsaturated fatty acids effects on immunomodulation

Polyunsaturated fatty acids (PUFAs) are essential fatty acids that contain more than one double bond in their backbone. PUFAs are divided into two main groups: omega-3 and omega-6. The structural chemical difference between the two groups is represented by the location of the cis double bonds (215). Together with cholesterol, PUFAs are essential for cell membrane integrity, development and maintenance in the homeostasis of cell function. Moreover, they are used by certain structures in cells and they stimulate cell proliferation (216). Calder and Grimble demonstrated that the changes in fatty acids’ membrane composition can affect membrane fluidity, mostly due to their modification in the enzymes’ affinity for substrates, which can change the signaling pathways. In this way, the sensitivity of the immune function can be modified (217). The most representative polyunsaturated fatty acids are eicosapentaenoic acid (EPA), alpha-linolenic acid (ALA), and docosahexaenoic acid (DHA), all defined as omega-3 fatty acids (215). They are very intensively studied since they are involved in many essential vital activities and more interestingly in immunomodulation pathways. In addition, the ALA is important due to the fact that it is a precursor of other fatty acids (218). Omega-3 PUFAs have a role in immunomodulation by decreasing pro-inflammatory eicosanoids. They represent a substrate for AA cascade enzymes, in this way certain prostanoids and leukotrienes are produced. Some lipid mediators such as maresins have omega-3 PUFAs as precursors. They promote the ending of the inflammatory process (219). In human breast cancer cells ALA produce inhibition of cell proliferation and activate apoptosis (220). In diabetic rats, ALA increases insulin sensitivity and restored lipid and glucose metabolic abnormalities (221). ALA is considered essential because it cannot be synthesized by the human body. In these regards, from the omega-6 group, an essential is considered linoleic acid (LA). Following LA ingestion, this fatty acid is quickly converted into arachidonic acid (ARA), which is responsible for the fluidity as well as the flexibility of the cell membrane. Free ARA is involved in the modulation of ion channels, enzymes, and receptors through stimulation or suppression of their function (222). Free unesterified ARA exerts antitumoral activity *in vitro* as well *in vivo*. It can be used as an anti-cancer drug. (223). Moreover, ARA can cause the death of tumor cells through the suppression of proliferation determining in this way, the death via stimulation of neutral sphingomyelinase (nSMase) mechanism (224).

Omega-3, as well as omega-6, participate in immunomodulation. According to Simonetto et al. (225) they have antagonist effects. Omega-3 from the PUFAs group is involved in anti-inflammatory reactions through the inhibition of ARA from the membrane, which is the main precursor for pro-inflammatory eicosanoids (225). They are capable to modulate immune and inflammatory responses through intensity and duration. On the one hand, pro-inflammatory effects are linked to fever, vasodilatation and intensification of pain. On the other hand, they could have anti-inflammatory effects by blocking natural killer activity and lymphocyte proliferation. Also, they are capable to inhibit IL-6, IL-2, and TNF-α (217). However, most importantly is the ratio between the 2 groups of PUFAs. Simopoulos, tried to shade light in this regards and proposed that a low omega-6/omega-3 ratio in women is responsible for the decrease in breast cancer risk. She additionally concluded that a lower ratio is associated with a general decrease in very common chronic diseases in the Western society (226).

## Conclusion and future perspectives

### The nutrients which have major effects on normal immune cell function and immune homeostasis

As mentioned throughout all this review, there is a strong and dynamic link between nutrition and immune function, as a direct consequence of the modulation of the immune function through the pro-inflammatory and anti-inflammatory effects of certain nutrients including cholesterol who exerts a crucial impact in these complex biological settings and holds a great capacity to regulate immune function, tightly related to its concentration. Certain micronutrients mentioned in this review: A, B1, B2, B3, B12, C, and D vitamins and minerals such as Zinc, and Selenium affect innate as well as adaptive immunity specifically through genetic, biochemical, and signaling pathways. All these may be translated into the modulation of proliferation, cell division, cell mobilization, and physiology of immune cells.

Additionally, we provide evidence that some macro-nutrients such as tryptophan, arginine, cholesterol and PUFAs may be involved in the prevention and therapy of some immune-related diseases. Also, is very important to note that some vitamins such as A and D are fat soluble (227). That is why when we consume fat-free (light) products, we are practically deprived of fat-soluble vitamins and the immune function can be affected. So, western diets should contain the accurate class of healthy fats, such as PUFAs, in a correct ratio, otherwise the edible products become poor in nutrients. A good example is the Mediterranean diet. In addition, the fats are much more satiating and give food a much better taste (228).

### The nutrients implicated in inflammation and immunopathologies

We highlight the difference in response to micro- and macro nutrients between healthy and sick population. We provided evidence that the response in pathophysiologic stages are very different to normal physiologic stages additionally to interindividual variations. As a result, the immune response is different and variable (11, 229, 230). We also presented some evidences and speculations on the roles of some vitamins, as well as certain amino acids, in cancer patients, due to their involvement as cofactors in proliferation and energy-related pathways finally leading to the development of tumor cells. We foresee that further research needs to be done in order to clearly distinguish the possible oncogenic effects of thiamin, cobalamin, and arginine (58, 66).

We additionally provide evidence that the inflammatory responses in general, and the changes in immune functions are modified by the lack of an accurate cholesterol metabolism. The alterations in the cholesterol biosynthetic pathway may have both positive and negative immune health-related repercussions. Alterations in the cholesterol biosynthetic pathway may directly impinge and interfere with antimicrobial responses, as well as with antiviral effects (192). Thus, an immediate action is required in order to adjust the cholesterol metabolism.

### Perspectives on the role of nutrients in ameliorating severity of specific inflammatory diseases, autoimmunity

Moreover, we refer to the bioavailability of macro- and micro nutrients from food. We ask whether foods contain enough amounts of macro- and micro nutrients. Does the soil and then the foods today still have the same nutritional value as before for example in fruits and vegetables? These led us to question under what conditions can artificial supplementation with macro or micronutrients be done? And how should be done? Should they be taken alone or as a complex? The question is the synergistic and complementary action of taking supplements of vitamin complexes, results in a better or worst outcome? We surmise that the administration of nutrients (micro and macro) would exert distinct effects on each person. We know that each individual is different, and thus their immune responses will differ from each other. To add more complexity, we referred also to the absorption capability of nutrients in the different compartments of the digestive system. From all the information provided above we can establish that malnutrition and/or supplementation strongly affect the immune system.

We finally provided evidence that for each stage of the immune process, both micro and macronutrients are needed for the proper functioning of this important system.

## Author contributions

BS and CM wrote the manuscript after a rigorous investigation, interpretation, systematization, and conceptualization of current data. Both authors agreed to publish the present manuscript, contributed to the article, and approved the submitted version.
